# Supplementary material for: Structure of the GOLD-domain seven-transmembrane helix protein family member TMEM87A
Source: eLife. 2022 Nov 14;11:e81704. doi: 10.7554/eLife.81704 (PMC9711517; doi:10.7554/eLife.81704)
Supplement: Supplementary file 1. [file elife-81704-supp1.docx]

| **Data collection** | **TMEM87A** |
| --- | --- |
| PDB | 8CTJ |
| EMDB | 26992 |
| EMPIAR | 11045 |
| Total movies # | 7060 |
| Selected movies # | 5898 |
| Magnification | 36,000 x |
| Voltage (KV) | 200 |
| Electron exposure (e^-^/Å^2^) | 50 |
| Frame # | 50 |
| Defocus range (um) | -0.6 to -1.8 |
| Super resolution pixel size (Å^2^) | 0.5575 |
| Binned pixel size (Å^2^) | 1.115 |
| **Processing** |  |
| Initial particle images (no.) | 2,674,406 |
| Final particle images (no.) | 138,217 |
| Map resolution Masked (Å, FSC = 0.143) | 4.7 |
| Symmetry imposed | C1 |
| **Refinement** |  |
| Model resolution (Å, FSC = 0.143 / FSC = 0.5) | 3.9 / 5.9 |
| Map-sharpening B factor (Å^2^) | -421.5 |
| Composition |  |
| Number of atoms | 3312 |
| Number of protein residues | 401 |
| Number of ligands | 1 |
| R.m.s. deviations |  |
| Bond lengths (Å) | 0.004 |
| Bond angles (Å) | 0.855 |
| Validation |  |
| MolProbity score | 1.64 |
| Clashscore | 5.98 |
| Ramachandran plot |  |
| Favored (%) | 95.44 |
| Allowed (%) | 4.56 |
| Disallowed (%) | 0 |
| Rotamer outliers (%) | 0 |
| Mean B factor (Å^2^) |  |
| Protein | 153.9 |
| Ligand | 158.59 |
